# The effect of paraoxonase 1 (PON1) gene polymorphisms T(-107)C and L55M and diet composition on serum PON1 activity in women

**DOI:** 10.20945/2359-3997000000416

**Published:** 2021-11-11

**Authors:** Bianka Machado Zanini, Leticia Burkert, Fabiola Goettem dos Santos, Michal M. Masternak, José Augusto Crespo-Ribeiro, Carlos Castilho Barros, Sandra Costa Valle, Augusto Schneider

**Affiliations:** 1 Universidade Federal de Pelotas Faculdade de Nutrição Departamento de Nutrição Pelotas RS Brasil Departamento de Nutrição, Faculdade de Nutrição, Universidade Federal de Pelotas, Pelotas, RS, Brasil; 2 University of Central Florida Burnett School of Biomedical Sciences College of Medicine Orlando FL USA College of Medicine, Burnett School of Biomedical Sciences, University of Central Florida, Orlando, FL, USA; 3 Universidade Federal de Pelotas Faculdade de Medicina Departamento de Ginecologia Pelotas RS Brasil Departamento de Ginecologia, Faculdade de Medicina, Universidade Federal de Pelotas, Pelotas, RS, Brasil

**Keywords:** SNPs, nutrigenetics, HDL, cardiovascular disease, atherosclerosis

## Abstract

**Objective:**

The aim of this study was to evaluate the serum activity of PON1 in women according to SNPs L55M and T-107C and diet composition.

**Materials and methods:**

Blood and serum samples from 26 women were used. DNA extraction, PCR and digestion with restriction enzymes of the PCR fragment were performed for genotyping the PON1 SNPs T-107C and L55M. Serum PON1 activity was measured in a single time point. Patients completed the semi-quantitative food frequency questionnaire and diet composition was estimated.

**Results:**

Genotypic distribution for L55M SNP was 56% for the LL genotype, 32% for LM and 12% for MM; for the PON1 C(-107)T SNP it was 28% for the TT genotype, 41% for CT and 31% for CC. Individuals with C and L alleles had higher serum PON1 activity. Combining the two SNPs, we observed that individuals carrying the LL and CC genotypes had twice the activity of carriers of the TT and MM genotypes. Considering food intake, no significant difference was observed between genotypes and intake levels.

**Conclusion:**

PON1 T(-107)C and L55M SNPs exert a strong effect on serum PON1 activity in an additive manner and are more important than diet to predict serum PON1 activity.

## INTRODUCTION

The paraoxonases (PON) 1, 2 and 3 are part of a gene family consisting of three adjacent genes located in chromosome 7 in humans (1). PON1 is the most studied member of this family, and it is found in serum associated with high density lipoproteins (HDL) (2,3). PON1 is secreted by the liver and protects lipoproteins and cell membranes against lipid peroxidation, conferring antioxidant activity to HDL and inhibiting the oxidation of low-density lipoproteins (LDL) (4). PON1 activity reduces as the individual ages, which is possibly related to increased oxidative stress (5,6). Conflicting data also show that there are variations between genders regarding serum PON1 activity (7,8). In addition to age and gender, other factors such as tobacco use, diabetes type 1 and 2, hypertension and hypercholesterolemia seem to reduce PON1 activity (9-11).

The main modifiable risk factors for cardiovascular disease (CVD) are smoking, high cholesterol and hypertension (12). However, serum PON1 activity is lower after myocardial infarction (13), suggesting it is an important risk factor to the development of CVD. Low PON1 levels appear to be associated with the formation of atherosclerotic plaque, and thus, the risk of developing CVD becomes higher (7). In this sense, diet composition has been shown to also be a strong modifier of PON1 activity in rodents (14,15). In humans, studies have shown that intake of saturated (SFA) and monounsaturated fatty acids (MUFAs) increase PON1 activity, while polyunsaturated fatty acids (PUFA) reduce PON1 activity (16). However, other have shown that intake of dietary fats did not affect PON1 activity (17) and even that consumption of PUFAs can increase PON1 activity (18). Therefore, results regarding the effect of dietary fats on PON1 activity are controversial. In this context, further studies are necessary to elucidate the association between diet and PON1 activity.

It is well known that single nucleotide polymorphisms (SNP) in the PON1 gene are also directly associated with the expression and activity of PON1 (19). Among the SNPs described in the PON1 gene, two are quite common and are located on the coding region. One results in the exchange of a glutamine for an Arginine at position 192 (Q192R) and the other changes a leucine to a methionine at position 55 (L55M) (20). Individuals with 55 LL genotype have a higher serum PON1 activity than individuals with genotype 55 MM (21-23) depending on diet composition (24). In addition, SNPs have also been identified in the promoter region of the gene, located at positions -107/108, -126, -160/162, -824/832 and -907/909 (24). The SNP known as T(-107)C has an effect on gene expression and consequently on serum PON1 activity. The PON1-107 CC genotype is associated to higher serum PON1 activity (25) and is also influenced by diet composition (25).

Serum PON1 activity in females is higher than in males (26) as estradiol has been shown to enhance PON1 activity independent of liver synthesis (27). Despite this, our previous study showed no difference between pre and post-menopausal women regarding serum PON1 activity or using contraception (28). Therefore, better understanding regulation of serum PON1 activity in women can help improve prevention of chronic diseases. Since PON1 has a protective effect against the development of CVD and that several factors, as SNPs and diet composition, have a direct impact on serum PON1 activity, we aimed to investigate the association of serum PON1 activity in women with the PON1 55 and -107 SNPs and diet composition.

## MATERIALS AND METHODS

### Population and sample collection

Blood samples from a cross-sectional study involving 26 healthy patients, aged over 18 years who attended at the Gynecology Outpatient Clinic from the School of Medicine and signed the informed consent form were used. Blood samples were collected by venipuncture after a 12-hour fasting. Part of the blood sample was collected with EDTA for DNA extraction and the remaining was centrifuged for serum harvesting to measure PON1 activity. The study was approved by the Federal University of Pelotas Ethics Committee CAAE number 30257514.5.0000.5317.

### Anthropometric measurements and food intake

Anthropometric measurements were taken by measuring body mass (kg), height (m) to estimate the body mass index (BMI). BMI was calculated by applying the formula BMI = body mass/height^2^ and interpreted according to the World Health Organization (29). For estimating intake, we used a semi-quantitative food frequency questionnaire (FFQ) referring to the last 12 months of consumption (30). The participants were guided by a nutritionist to fill the forms. The questionnaire consisted of 78 food items (31). The data from FFQ was computed in spreadsheets (Microsoft Excel, 2016) for the conversion of the measurements according to the Brazilian Food Composition Table (TACO). From this we estimated individual intake of calories (kcal/d), total dietary fat (g/d), vitamin A and C, zinc, SFA, MUFA, PUFA intake and as a percentage of the total daily energy intake (%), omega-3 (n-3; g/d) and -6 (n-6;g/d) and the ratio of n-6/n-3.

### DNA extraction, polymerase chain reaction (PCR) and enzyme digestion

For DNA extraction, whole blood samples were used. DNA was extracted according to a previous published protocol (32). Quantitation of final DNA solution was performed on a NanoDrop® equipment and standardized for 100 ng/µL of DNA for use in the PCR reaction.

The L55M and T(-107)C SNPs were obtained by PCR followed by restriction enzyme digestion according to Rantala and cols. (24) and Campo and cols. (8), respectively. For amplification of the coding region of the PON1 gene where the L55M SNP is located, the GOTaq® (Promega, Madison, WI, USA) PCR mix was used with 10 nM of the forward primer (GAAGAGTGATGTTATAGCCCCAG), 10 nM of the reverse primer (ACTCACAGAGCTAATGAAAGCCA) and 400 ng of DNA. For amplification samples were incubated in a thermocycler for 5 minutes at 95 °C and subsequently submitted to 35 cycles of 95 °C for 45 seconds; 61 °C for 45 seconds; 72 °C for 1 minute and a final cycle of 72 °C for 10 minutes. PCR products were digested with the restriction enzyme NlaIII (1 IU, New England Biolabs, Ipswich, MA, USA) using the CutSmart™ buffer (10x). Samples were incubated at 37°C for two hours. After digestion, products were submitted to SYBR® Safe (Life Technologies, Carlsbad, CA, USA) stained (3%) agarose gel electrophoresis with a 100 bp sample marker. Digestion resulted in 127- and 42-bp fragments for the PON1 55 M allele and a non-digested 169-bp fragment for the PON1 55 L allele.

For the amplification of the promoter region of the PON1 gene where the T(-107)C SNP is located the PCR was performed using the following primers forward (AGCTAGCTGCGGACCCGGCGGGGAGGaG) and reverse (GGCTGCAGCCCTCACCACAACC). PCR conditions were identical to above described except the annealing temperature was 67 ºC. The lower-case letter in the forward primer indicates a mismatch that introduces a restriction site for the BsrBl endonuclease (New England Biolabs), as there is no specific restriction site cutting the original DNA sequence. After digestion for 2 hours at 37 ºC with 1 IU of the enzyme, samples were submitted to agarose gel electrophoresis as described before. The PON1-107 C allele was identified by the 28 and 212bp fragments in the gel, while the T allele resulted in the non-digested 240 bp fragment.

### Serum PON1 activity

For analysis of serum PON1 enzyme activity serum samples were diluted 3:1 in Tris/HCl buffer (20 mmol/L Tris/HCl, pH 8.0, containing 1 mmol/L of CaCl_2_). For the activity assay the diluted serum was added to the Tris/HCl buffer containing phenyl acetate (4 mmol/L). Absorbance was measured in a spectrophotometer (FEMTO, São Paulo, SP, Brazil) at a wavelength of 270 nm for 60 seconds. PON1 activity was expressed in U/mL, based on the phenol extinction coefficient.

### Statistical analysis

Data was analyzed using SAS University Edition (SAS, Cary, NC, USA). Age and BMI were used as co-factors in all analysis. The MIXED procedure was used to test the effect of SNPs on PON1 serum activity, in addition the GLM procedure was run to test the linear effect of favorable alleles. Favorable genotypes for each SNP in the PON1 gene (-107 and 55) were combined to observe their additive effect on PON1 activity (-107TT/55MM = 0 and -107CC/55LL = 4). Diet intake was classified according to the median intake of the group. For the multivariate model, a stepwise logistic regression procedure was performed to identify independent variables that contributed the most for predicting serum PON1 activity (High vs. Low based on median). A backward selection technique was used to eliminate covariates that did not contribute to the model. A significance level of 0.3 or above was used to remove covariates from the multivariable model, and a value of 0.3 or less was used to include variables. Data are presented as mean ± standard error of mean. P values lower than 0.05 were considered as significant.

## RESULTS

The anthropometric and biochemical characteristics evaluated are presented in Table 1 From this population 27% (n = 7) were postmenopausal women. The allele frequency for the PON1 L55M SNP in the population analyzed was higher for the L allele (69.2%) and for the PON1 T(-107)C the C allele was more prevalent (53.8%). In this study we observed for the L55M SNP the genotypic distribution of 56% for the LL genotype, 32% for LM and 12% for MM. Regarding the PON1 C(-107)T SNP genotype distribution, it was 28% for TT genotype, 41% for TC and 31% for CC. For the combined frequency, the distribution was 7.7%, 11.5%, 26.9%, 34.6% and 19.2% for carriers of 0, 1, 2, 3 and 4 for favorable C and L alleles, respectively.

**Table 1:** Anthropometric, biochemical and dietary composition characteristics women participating in the study, Pelotas, RS, Brazil (n = 26)

Parameter	Value (±SEM)
Age, years	42.3 ± 2.7
BMI	26.7 ± 0.8
Paraoxonase, U/mL	99.4 ± 4.7
HDL, mg/dL	57.5 ± 2.7
LDL, mg/dL	125.1 ± 6.7
Cholesterol, mg/dL	213.8 ± 7.4
TAG, mg/dL	156.2 ± 14.4
Glucose, mg/dL	87.6 ± 2.2
Diet values		
Calories, kcal/d	2,194.8 ± 112.4
Fat, g/d (%CI)	57.5 ± 3.5 (23.8 ± 1)
SFA, g/d (%CI)	21.6 ± 1.3 (16.5 ± 0.9)
MUFA, g/d (%CI)	14.4 ± 1 (11.0 ± 0.6)
PUFA, g/d (%CI)	12.4 ± 1.2 (8.9 ± 0.4)
ω-3, g/d	0.7 ± 0.1
ω-6, g/d	11.3 ± 1.1
ω-6: ω-3	16 ± 0.8

BMI: body mass index; HDL: high density lipoprotein cholesterol; LDL: low density lipoprotein cholesterol; TAG: triglycerides; CI: caloric intake; SFA: saturated fatty acids; MUFA: mono unsaturated fatty acids; PUFA: poly unsaturated fatty acids; ω-3: ômega 3; ω-6: ômega 6.

**Figure 1:**
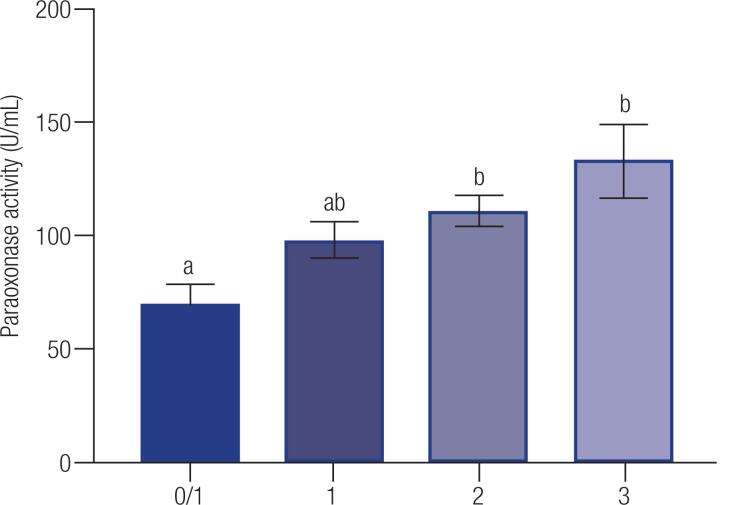
Paraoxonase 1 activity (U/mL) for the combined SNPs T(-107)C and L55M in women (n = 26). Data are presented as means ± SEM. Different letters indicate significant difference at P < 0.05.

The overall serum PON1 activity was 99.5 ± 4.7 U/mL in the analyzed population. Both SNPs had a significant effect on PON1 activity, as women with C and L alleles had higher serum PON1 activity (Table 2). Table 2 also shows HDL, LDL, cholesterol, triglycerides and glucose concentrations, which were not different for both -107 and 55 genotypes (P > 0.05). When combining both SNPs favorable alleles (C and L) we observed a strong effect on serum PON1 activity (Figure 1, P < 0.05). There was a linear effect of increasing number of favorable C and L alleles (P = 0.007).

**Table 2:** PON1 activity and serum parameters for each genotype for the two SNPs in women participating in the study, Pelotas, RS, Brazil (n = 26)

	PON -107			P-value	PON 55			P-value
	TT	CT	CC		MM	LM	LL	
PON	64.5 ± 16.0^a^	93.1 ± 5.2^a^	128.0 ± 9.0^b^	0.01*	66.3 ± 10.8^a^	108.5 ± 7.5^b^	109.2 ± 8.8^b^	0.004*
HDL	53.8 ± 16.6	56.7 ± 5.7	57.4 ± 9.3	0.96	55.8 ± 9.8	59.6 ± 7.6	53.4 ± 7.9	0.93
LDL	120.3 ± 38.3	128.3 ± 12.5	146.4 ± 21.5	0.72	123.0 ± 22.9	128.5 ± 16.0	143.6 ± 18.7	0.74
COL	206.6 ± 89.6	246.6 ± 29.2	324.2 ± 50.4	0.41	203.0 ± 55.7	290.7 ± 38.8	261.3 ± 45.3	0.27
TAG	77.3 ± 76.0	146.5 ± 24.8	182.1 ± 42.7	0.54	73.0 ± 36.6	194.6 ± 25.5	138.9 ± 29.7	0.58
GL	73.4 ± 9.6	86.4 ± 3.1	95.3 ± 5.4	0.13	84.0 ± 7.0	89.6 ± 4.9	86.8 ± 5.7	0.58

PON: paraoxonase; HDL: high density lipoprotein cholesterol; LDL: low density lipoprotein cholesterol; COL: cholesterol; TAG: triglycerides; GL: glucose.

* Different superscript letters indicate statistical difference.

**Figure 2:**
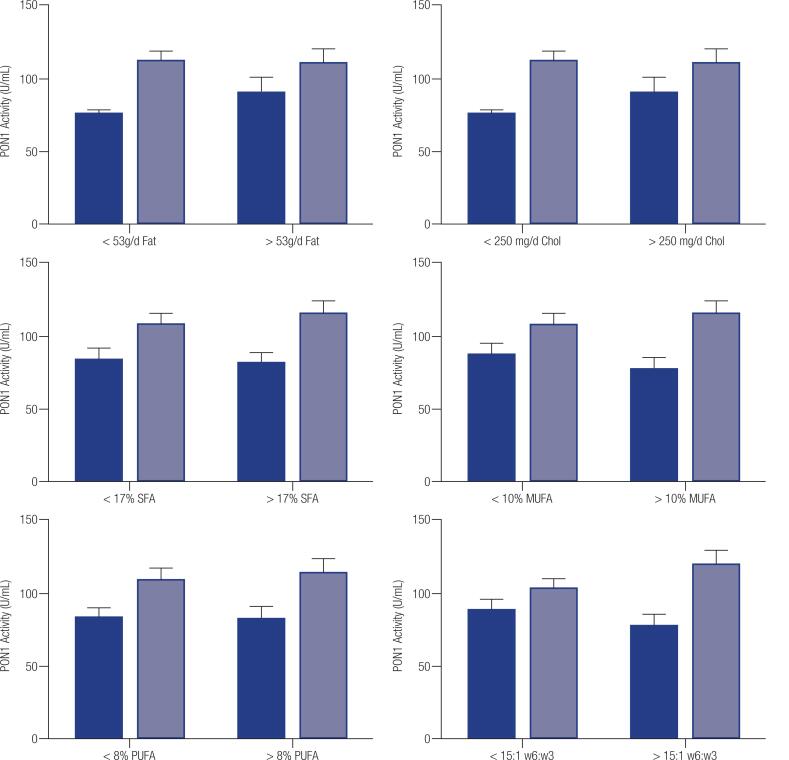
Analyze of combined L55M/T(-107)C SNPs and diet intake on serum PON1 activity (n = 26). Black bars are from genotypes 0, 1 and 2 and white bars for genotypes 3 and 4. The X axis indicate groups of intake below and above the median. Data are presented as means ± SEM.

When we associated the dietary profile to PON1 activity, regardless of genotype, no significant difference was observed between women with low and high intake for total fat, cholesterol and of each type of fatty acid (data not shown; P > 0.05). In addition, when combining the effects of diet and genotype no significant effects were observed either (P > 0.05, Figure 2).

The multivariate logistic regression model was used to identify which variables contributed the most to the serum PON1 activity in this group of women. Only zinc (P = 0.01), MUFAs (P = 0.03), cholesterol (P = 0.18), age (P = 0.10), omega 3 (P = 0.07) and PON1 T(-107)C SNP (P = 0.03) remained in the final model in this order, respectively.

## DISCUSSION

PON1 provides HDL antioxidant activity and inhibits the oxidation of low-density lipoproteins, playing an important role in prevention of cardiovascular diseases. In this study we found that PON1 T(-107)C and L55M SNPs had a strong association with PON1 activity in women. Interestingly, the effect was even stronger when we combined the favorable alleles for each SNP in a single analysis. Carriers of 4 favorable alleles (CC plus LL genotype) had twice activity of PON1 compared to carriers of 0 or 1 favorable allele. Based on the activity levels of PON1, these findings may be important in the diagnosis of women who are more susceptible to developing cardiovascular diseases, taking into account the association between serum PON1 levels and CVD (13).

For the L55M SNP, carriers of the L allele had higher serum PON1 activity compared to carriers of the M allele, as previously observed by others (21,23,33). The same was observed for the T(-107)C SNP, where women carrying the C allele had higher serum PON1 activity, in agreement with previous studies (25). However, there are no records in the literature of studies that made a combined evaluation of both SNPs T(-107)C and L55M. Through this work we present for the first time the distribution of these genotypes in a population of women. No studies for the combined L55M and T(-107)C effects were found for men either. We observed that more than half of the population studied (53%) carried 3 or 4 favorable alleles. These women had almost double the serum PON1 activity than carriers of 0 or 1 favorable alleles. We observed that the effects of the combined SNPs on PON1 activity were more informative than when individually analyzed. Regarding the frequency of the studied SNPs, 72% of women carried the PON1 L55M L allele. Expected frequency for the overall population (men and women) is 82%, while in the American population is 79% and for the European population is 64% (32), in agreement with our findings. Regarding the PON1 T(-107)C SNP, the C allele frequency was 53.8% in our study. Typically expected frequency for overall population is 65% (men and women), while for American populations is 56% and for European population is 51%, also in agreement with our current findings (32).

Despite the clear effect of the genotype on PON1 activity, we did not observe any direct modulation of the diet on PON1 activity, such as excessive consumption of total fat, SFA, MUFA, PUFA and cholesterol. This suggest that, in the female population, the presence of the favorable allele remains more important than diet to predict serum PON1 activity. Several studies have shown that diet composition influences serum PON1 activity. Diets rich in SFA and PUFA were associated to changes in PON1 activity, but there is controversy regarding the influence of an atherogenic diet on PON1 levels (16-18) Overall, it was shown before that the contribution of diet to PON1 activity is less than 10% (16). Even when we tried to evaluate the combined genotype effect with the diet, no significant differences were found. We have to acknowledge that the number of samples in our study was small for further in-depth comparison. Others showed that the presence of the T(-107)C allele was associated with increased serum PON1 activity, however a high SFA diet reduced PON1 activity in women of the CC genotype (25). Nevertheless, even in this small samples we observed a strong direct genotype effect and no effect of the diet, again suggesting the more important role of SNPs in PON1 modulation.

When we evaluated serum PON1 activity based on median intake no effects were observed. This was the same when we tried to include in this analysis the combined genotype. However, in the multivariate analysis when testing the dietary and genetic factors that would be the most predictive of serum PON1 activity, we observed that dietary zinc, MUFAs, cholesterol and omega 3 intake as well as age play an important role. A previous study had found in a multivariate analysis including both sexes that genotype, age, sex and cholesterol intake were the most predictive of PON1 activity (16). Another similar study found that after genotype the most important predictors of serum PON1 activity were dietary fatty acids and cholesterol (34). Interestingly, a previous study showed that zinc was able to modulate PON1 activity having anti-inflammatory and antioxidant properties (35) in accordance to our current observation. Diets rich in MUFAs are known to increase serum PON1 activity (34), and the same was observed for cholesterol (34), which we also observed in this group of women. Regarding omega-3, studies are controversial, with some observing that serum PON1 activity was reduced in rats fed diet rich in fish oil (15), while in humans a study showed increased PON1 activity after omega-3 supplementation (36). Our previous study with observed that higher omega6:omega3 ratio increased PON1 activity (36), suggesting the ratio is more important than total intake. Regardless, our current findings are in line with previous evidence and suggest that dietary zinc, MUFA, cholesterol and omega-3 intake are important regulators of serum PON1 activity along with age and genotype in reproductive age women and can play a role in prevention of CVD.

We want to emphasize that this study used a small number of female patients only and results should be interpreted carefully. Results should be also interpreted in light of the limitation of the semi-quantitative FFQ for accessing daily intake of nutrients. Also, it should be taken into consideration that we did not access physical activity of subjects.

In conclusion, our data strongly support that SNPs T(-107)C and L55M have an effect on serum PON1 activity. Additionally, combining the favorable alleles for these SNPs increased the power to identify women with higher PON1 activity and may be a better approach regarding PON1 SNPs and serum activity. Dietary zinc, MUFA, cholesterol and omega-3 intake were shown to predictors of serum PON1 activity along age and genotype.
